# Stability of misoprostol tablets collected in Malawi and Rwanda: Importance of intact primary packaging

**DOI:** 10.1371/journal.pone.0238628

**Published:** 2020-09-02

**Authors:** Nhomsai Hagen, Thomas Bizimana, P. Claver Kayumba, Felix Khuluza, Lutz Heide

**Affiliations:** 1 Pharmaceutical Institute, Eberhard Karls University Tubingen, Tubingen, Germany; 2 School of Medicine and Pharmacy, College of Medicine and Health Sciences, University of Rwanda, Kigali, Rwanda; 3 East African Community Regional Centre of Excellence for Vaccines, Immunizations and Health Supply Chain Management (EAC RCE-VIHSCM), University of Rwanda, Kigali, Rwanda; 4 Pharmacy Department, College of Medicine, University of Malawi, Blantyre, Malawi; Bhagwan Mahvir College of Pharmacy, INDIA

## Abstract

Misoprostol is listed in the WHO essential medicines list and can be used for induction of labour, for prevention and treatment of post-partum haemorrhage, and for abortions. The compound is unstable, and substandard misoprostol preparations have been detected in low- and middle-income countries. We now investigated the stability of misoprostol tablets according to the international guidelines for stability testing of pharmaceutical products. Three brands (four batches) of misoprostol tablets were collected in Malawi and Rwanda: the originator product, a WHO-prequalified product, and a generic product without WHO prequalification. A further batch of the originator product was collected in Germany. To investigate the effect of damage to the primary packaging, additional blister strips of one sample were intentionally damaged with a needle and investigated in parallel. Samples were placed in stability chambers for six months at 40°C/75% relative humidity (RH) and at 25°C/60% RH. After 0, 1, 2, 3 and 6 months, misoprostol content was determined according to the International Pharmacopeia. At 40°C/75% RH, all samples showed a decline of misoprostol content, but four of the batches still remained within the pharmacopeial specifications, while one of the two batches of the generic product without WHO-prequalification showed a final content of 86.2% which is out of specifications. Damage to the primary packaging greatly decreased stability, resulting in a final content of only 48.2% of the declared misoprostol amount. At 25°C/60% RH all samples remained in specifications for six months, even the sample with the damaged blister. Dissolution of misoprostol remained in specifications of the pharmacopoeia for six months for all batches, except for the sample with damaged blisters stored at 40°C/75% RH. This study confirms that the stability of misoprostol tablets must be ensured by intact, good-quality primary packaging. Careful supplier qualification is required in the procurement process.

## Introduction

**Fig 1:**
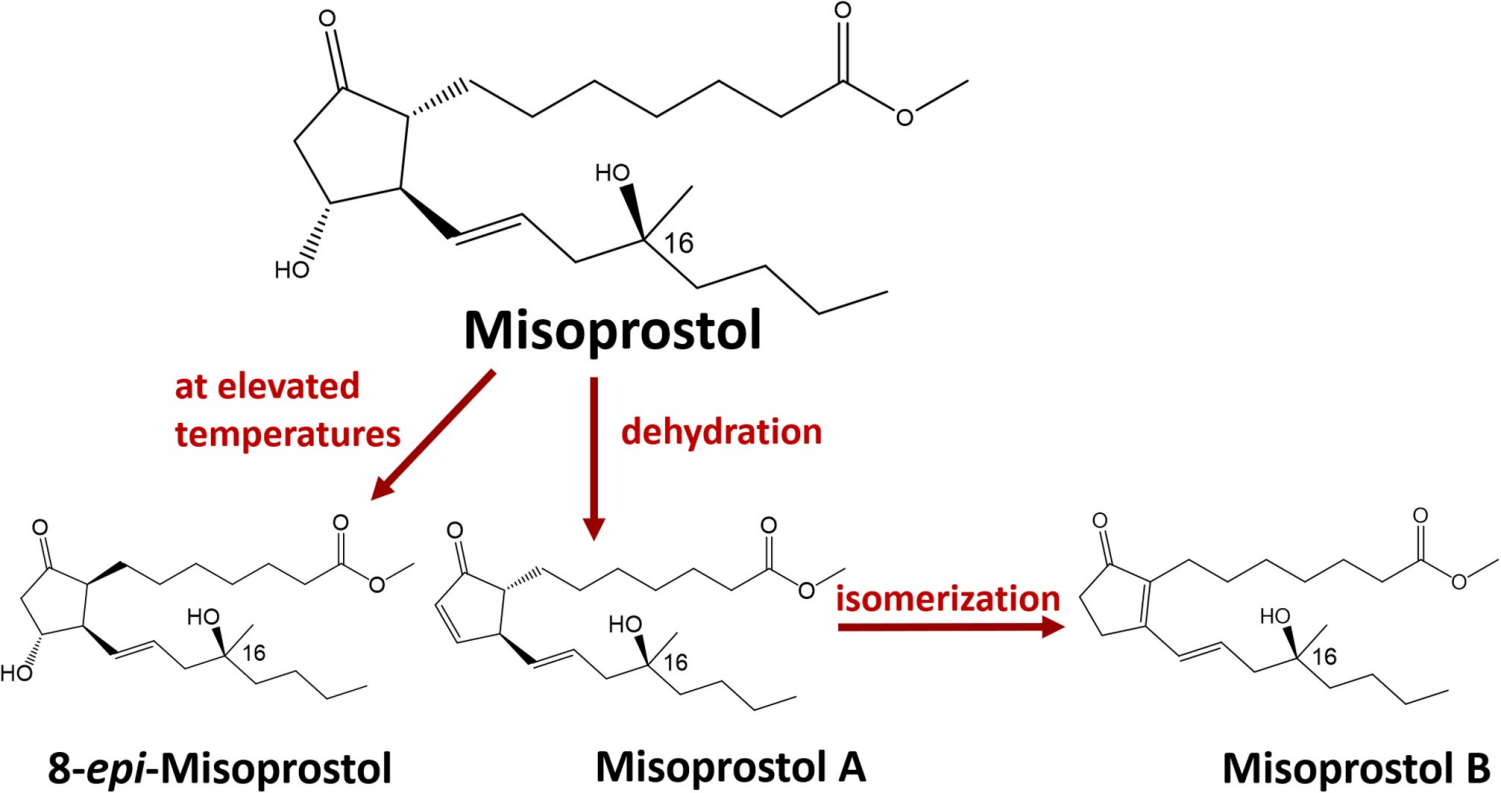
Structure of misoprostol and its typical degradation mechanisms. Commercial misoprostol is a mixture of the depicted structure at its epimer at C-16, as well as the enantiomers of both compounds. Corresponding stereoisomers are found for the degradation products.

Misoprostol (Fig 1), a prostaglandin analogue, is listed in the essential medicines list of the World Health Organization (WHO) [1] as a uterotonic and can be used for prevention and treatment of post-partum haemorrhage (PPH). PPH is the leading cause of maternal mortality in low-income countries. It is defined as a blood loss of 500 ml or more within 24 hours after giving birth [2]. Oxytocin, the medicine of first choice for prevention and treatment of PPH, is very sensitive to environmental conditions. It degrades at high temperatures and usually has to be stored at 2–8 °C [3–7]. This storage requirement can be challenging, especially in rural areas of low- and middle-income countries (LMICs) where infrastructure is poor and ambient temperatures are often high [8–12]. Indeed, several previous studies have shown that the quality of oxytocin, especially in LMICs, is often poor [3, 10–17].

Misoprostol is available in form of tablets which can be administered orally and which do not require refrigerated storage. Therefore, misoprostol tablets appear to offer an attractive alternative to oxytocin injections in the prophylaxis and treatment of PPH in places where no trained staff is available to administer medicines parenterally, or where appropriate storage conditions for oxytocin cannot be ensured [1, 18, 19]. Misoprostol is also used for the induction of labour, for treatment and prevention of ulcers induced by non-steroidal anti-inflammatory drugs (NSAIDs) and for abortions [1, 20]. Concerns about its latter use have motivated some countries to restrict its availability.

The instability of misoprostol has been known since decades [21]. The most important degradation reaction is dehydration, leading to misoprostol A (Fig 1). Toledo-Vasquez et al. [22] described that in aqueous solutions, this reaction follows first-order kinetics, and at 60°C and pH 7.66 results in a degradation of misoprostol with a half-life of only 8.8 hours. Dehydration is followed by the much slower isomerization to the resonance-stabilized misoprostol B [21, 22] (Fig 1). Epimerization of misoprostol at C-8 is a further degradation mechanism occurring at higher temperatures [21].

Pure misoprostol shows 50% degradation within two weeks at 55°C [22]. Fortunately, a dispersion of misoprostol in hydroxypropyl methylcellulose (HPMC) is much more stable, allowing the formulation of finished pharmaceutical products [21–24]. However, these misoprostol-HPMC dispersions have to be carefully protected from humidity, since the degradation velocity of misoprostol increases sharply when the water content of the dispersion exceeds 2% [24], and tablets containing misoprostol-HPMC dispersions have been shown to absorb water very quickly when stored without protection [23]. Importantly, Hall [25] demonstrated that plastic-aluminium blisters which are frequently used for the packaging of pharmaceutical products are grossly inadequate for ensuring the stability of misoprostol tablets. This study investigated 215 samples of misoprostol tablets of different age after manufacture, collected in Asia, Africa and Latin America. When one year or more had elapsed after manufacture, all samples packaged in plastic-aluminium blisters showed a misoprostol content below the limit specified by the International Pharmacopeia (90% of the declared amount). Double-sided aluminium blisters provided better protection, but one year or more after manufacture, even of those 28% were reported to show misoprostol contents below the pharmacopeial limit [25].

The quality of misoprostol preparations circulating in LMICs has received much less attention in the scientific literature than the quality of oxytocin. Besides the above-mentioned study by Hall [25], only two reports have been published: Anyakora et al. [11] investigated 166 samples of misoprostol tablets collected in health facilities in Nigeria. 33.7% of these were reported to fail the specifications of the International Pharmacopeia for misoprostol content, but the report did not specify how far these samples deviated from declared content. Our group recently investigated the quality of oxytocin and misoprostol samples collected in health facilities and drug outlets in Malawi. Out of the 30 misoprostol samples, five (17%) showed extreme deviations, containing only 12.7–30.2% of the declared content [12]. In the scientific literature, no systematic stability study of finished pharmaceutical products of misoprostol has been published so far.

During the registration of medicines, National Medicines Regulatory Authorities (NMRAs) examine the stability data provided by the manufacturers, usually they do not carry out independent experiments to confirm these data. The NMRAs of the EU member states, USA, Japan, Switzerland, Canada, Australia, Iceland, Liechtenstein and Norway are currently considered as “Stringent Regulatory Authorities” (SRAs) [26], and medicines produced under supervision of an SRA are usually regarded with trust in regard to their manufacturers’ specifications for quality and stability. Similar trust is given to medicines which have received “WHO Prequalification” status, based on information submitted by manufacturers to WHO and on inspections of the respective manufacturing sites by experts mandated by WHO [27, 28]. Among the medicine types which are currently eligible for WHO prequalification status are medicines for reproductive and maternal health, including oxytocin injections and misoprostol tablets. However, only a small part of the medicines circulating in LMICs are approved by a SRA or WHO-prequalified [29]. In the present study, we investigated different batches of three misoprostol preparations: one preparation which was produced in a country with an SRA, another one which was a WHO-prequalified product, and one preparation which was produced in a non-SRA country and was not WHO-prequalified.

Rules for stability testing of pharmaceutical products have been devised by the International Council of Harmonization of Technical Requirements for Registration of Pharmaceuticals for Human Use (ICH), most importantly guidelines Q1A-Q1F [30]. Obviously, the aim of testing is to confirm the stability of the product over the entire shelf-life, but testing durations of several years are difficult from an economic viewpoint. Therefore, so-called accelerated stability studies are permissible. They are carried out for shorter time periods but at higher temperatures and relative humidities. These studies allow to predict the shelf life of the products under given storage conditions, and their results are used to decide about the label statements regarding shelf life and storage requirements for the respective products. Guidelines by ICH [31] and by WHO [26] specify the precise conditions for accelerated stability studies. Obviously, these conditions are related to the climatic zone of the country where the medicine will be registered. Malawi is currently assigned to WHO climatic zone II (subtropical and Mediterranean), and Rwanda to zone IVa (hot and humid) [32].

In the present study, accelerated stability testing of misoprostol tablets collected in Malawi and Rwanda was carried out according to the ICH guidelines for stability testing of pharmaceutical products [31].

## Materials and methods

### Study design and ethical clearance

This study was designed observing the recommendations contained in the WHO guidelines on the conduct of surveys of the quality of medicines [33], the MEDQUARG guidelines [34], and the ICH and WHO guidelines for stability testing of pharmaceutical products [26, 31]. The College of Medicine Research and Ethics Committee (COMREC) in Malawi gave ethical clearance to conduct this study (Reference No. P.07/27/2215), as well as the Ministry of Health, Rwanda (Reference No. 20/1361/DGPHFIS/2018). Approval was also granted by the Malawi National Regulatory Agency (Pharmacy, Medicines and Poisons Board, PMPB).

### Sample collection

The brands of misoprostol tablets available at government medical stores and pharmaceutical wholesalers in Malawi and Rwanda during the time of sample collection (February-March 2018) were purchased by local researchers (F.K. and T.B.). If different batches of a certain brand were available, samples from each batch were purchased. From each brand and batch, 250 tablets were purchased. Samples were not collected from individual health facilities or private pharmacies, to minimize the influence of possibly incorrect storage conditions on the results of this study. Also, these facilities usually do not stock sufficient amounts of misoprostol tablets for stability testing. After purchase, samples were stored according to the manufacturers’ specifications. Temperature data loggers were kept with the samples until these were placed into the stability chambers for testing. The investigators hand-carried the samples from Malawi or Rwanda to Germany via airplane; this transport required less than 24 hours. Samples were pre-tested for their misoprostol content according to International Pharmacopeia. Those samples which were within specifications at the time of pretesting were included into the stability study.

### Storage in stability chambers

The samples were stored in their original primary packaging in stability chambers of Alpha-Pharma-Service GmbH, Heilbronn, Germany, for six months (April-October 2018). Conditions were chosen in accordance to ICH and WHO guidelines for stability testing of pharmaceutical products [26, 31]. Accelerated stability testing was carried out at 40°C +/- 2°C and 75% +/- 5% relative humidity (RH). Samples stored at 25°C +/- 2°C and 60% +/- 5% RH, i.e. at the ICH conditions for long-term testing of non-refrigerated products, climatic zone II, were investigated for comparison. After 0, 1, 2, 3, and 6 months, 20 tablets of each sample were removed from both chambers and analysed at Tuebingen University, Germany (by N.H. and T.B.).

### Sample analysis

Samples were visually inspected and subsequently tested for identity, assay and dissolution following the procedures described in the monograph for misoprostol tablets of the International Pharmacopeia 2017. Prior to the experiments, the methods were validated according to the International Pharmacopeia. Misoprostol was identified and quantified by high performance liquid chromatography (HPLC), using an Agilent Infinity 1260 II with binary pump and variable wavelength detector (Agilent Technologies, Santa Clara, CA, USA), with acetonitrile/water (45:55 V/V) as mobile phase, flow rate 1.5 ml/min, column ReproSil-XR 120 C18, 5 μm, 150 mm x 4.6 mm (Dr. Maisch GmbH, Ammerbuch, Germany), and UV detection at 200 nm. The injection volume was 100 μl for identity and assay, or 250 μl for dissolution. For identity and assay, five tablets were dissolved in 50 ml mobile phase; two independent experiments were carried out, and of the resulting solutions two aliquots each were analysed by HPLC, yielding four measurements for each sample. The HPLC autosampler was set to 4 °C to avoid misoprostol degradation. Samples showing additional peaks were also tested for related substances according to Kahsay et al. [35], using freshly prepared solutions from three tablets per sample and performing the tests without delay.

Dissolution was investigated with a dissolution tester PT-WS 610 (Pharma Test Apparatebau AG, Hainburg, Germany). Six tablets per sample were tested separately as described in the monograph for misoprostol tablets in the International Pharmacopeia, with 500 ml of water R as dissolution medium, a temperature of 37°C, and a rotating paddle with 50 revolutions per minute. Samples were withdrawn after 30 min through an in-line filter. Misoprostol reference standard Ph. Eur. (batch N° 3.0) was obtained from the European Directorate for the Quality of Medicines (EDQM), Strasbourg, France.

At each time point during the stability testing, 5-point calibration curves were prepared to assure linearity. Intermediate precision [36] was calculated from the data of the calibration curves of the reference standards, (see S2 Table).

### Statistical analysis

Statistical evaluation was done using JMP 14.2 (SAS GmbH, Heidelberg, Germany). Significance levels of differences were calculated using uni- and multivariate analysis of variance (ANOVA) and student´s t-test. Differences were considered significant when p< 0.05.

## Results

### Overview of investigated misoprostol samples

In this study, misoprostol samples were investigated which were offered by government medical stores and private wholesalers in Malawi and Rwanda, i.e. preparations which reflect the actual quality and stability of medicines distributed in these two African countries. In February and March 2018, the local investigators (F.K. and T.B.) enquired about the available brands and batches of misoprostol tablets. Samples were then purchased from private wholesalers using a mystery shopper approach (i.e. ordering with the help of a licensed pharmacy shop), and from government medical stores in an overt approach. If different batches of a certain brand were available, samples from each batch were purchased. In Malawi, certain brands of misoprostol tablets had recently been withdrawn from the market [12], and only one single brand was offered for sale at the time of sample collection, in form of two different batches; both batches were purchased. In Rwanda, three brands were offered at the time of sample collection (only a single batch of each brand), and each brand was purchased. However, one of these brands showed an insufficient API content already in pre-testing, and had to be excluded from the subsequent investigation. The Rwanda Food and Drug Authority (RFDA) was alerted about this substandard brand. Further investigations initiated by RFDA confirmed our findings, and effective from February 2019, RFDA recalled the substandard brand from the Rwandan pharmaceutical market. As a comparison, the originator brand (Cytotec^®^) was purchased through the pharmacy of the University Hospital Tuebingen. This is the brand most commonly used in Germany. A different batch of this originator brand had also been purchased in Rwanda.

Therefore, as shown in Table 1, three brands (total 5 batches) were included into the stability testing. The originator brand had been produced in the UK, i.e. in a country with an SRA. One further brand represented a WHO-prequalified product, manufactured in India. The third brand was not WHO-prequalified, and had also been produced in India.

**Table 1:** Investigated misoprostol samples. All samples represented tablets with a stated content of 200 μg/ tablet.

Collected in:	Collected at:	Stated manufacturer (and brand name)	Country of manufacture	Mfg. date/Exp. date	Batch number	Stated shelf life	Stated storage requirements	Primary packaging	Stated excipients / formulation	Prequalifi-cation status
Malawi	wholesaler	**Fourrts (India) Laboratories Pvt. Limited** (KONTRAC 200)	India	Jun 17/ May 19	E0571	2 years	Below 30°C in a dry place. Protect from light	alu/alu blister	none declared	none
	Central Medical Stores Trust			Feb 17/ Jan 19	D2205					
Rwanda	government district pharmacy	**Acme Formulation Pvt. Ltd.** (Ace Miso)	India	Sep 16/ Aug 18	ACE160963	2 years	Below 30°C protected from light	alu/alu blister	misoprostol as HPMC dispersion (1%), excipients q.s.	WHO-PQ
	wholesaler	**Piramal Healthcare UK Limited** (Cytotec)	UK	Jul 17^1^/ Jun 20	B17173	3 years^1^	none	alu/alu blister	misoprostol-HPMC dispersion (1%), cellulose (microcrystalline), sodium carboxymethyl- amidon, hydrogenated castor oil	SRA
Germany	pharmacy of Tuebingen university hospital			Feb 17^1^/ Jan 20	B16131		Below 30°C protected from humidity		cellulose (microcrystalline), HPMC, sodium carboxymethyl-amidon, hydrogenated castor oil	

HPMC: hydroxypropyl methylcellulose. WHO-PQ: WHO-prequalified product. SRA: produced in a country with stringent regulatory authority.

^1^ information not stated on the packaging, but obtained from internet data bases [20, 37].

Surprisingly, packaging and leaflet of the originator brand collected in Rwanda did not state any storage requirements, while packaging and leaflet of the same brand collected in Germany stated that storage below 30°C was required. The same storage temperature was stated by the manufacturers of the two other brands. The stated requirements for protection from light and humidity were somewhat different between the brands (Table 1). All samples had double-sided aluminium blisters as primary packaging. Pre-testing for content of misoprostol showed that these samples were within the specifications of the International Pharmacopoeia at the beginning of the stability study.

The three manufacturers were contacted and were requested to confirm the authenticity of the samples. The two manufacturers from India confirmed that label information and appearance of the samples conformed to their products. The latest response from Pfizer (the marketing authorization holder)/Piramal was that they were still investigating this matter.The declared shelf life of the investigated brands was either two or three years. While four of the five investigated batches remained within their shelf life during the entire duration of the study, the WHO-prequalified product by Acme expired during the stability testing. Nevertheless, it was found to remain within specifications during the entire testing period, even after storage for six months at 40 °C and 75% RH (see below).

Table 1 also shows the stated excipients for the investigated preparations. Notably, for the product Kontrac 200^®^ which was neither SRA-approved nor WHO-prequalified, no excipients were declared at all.

### Accelerated stability study

**Fig 2:**
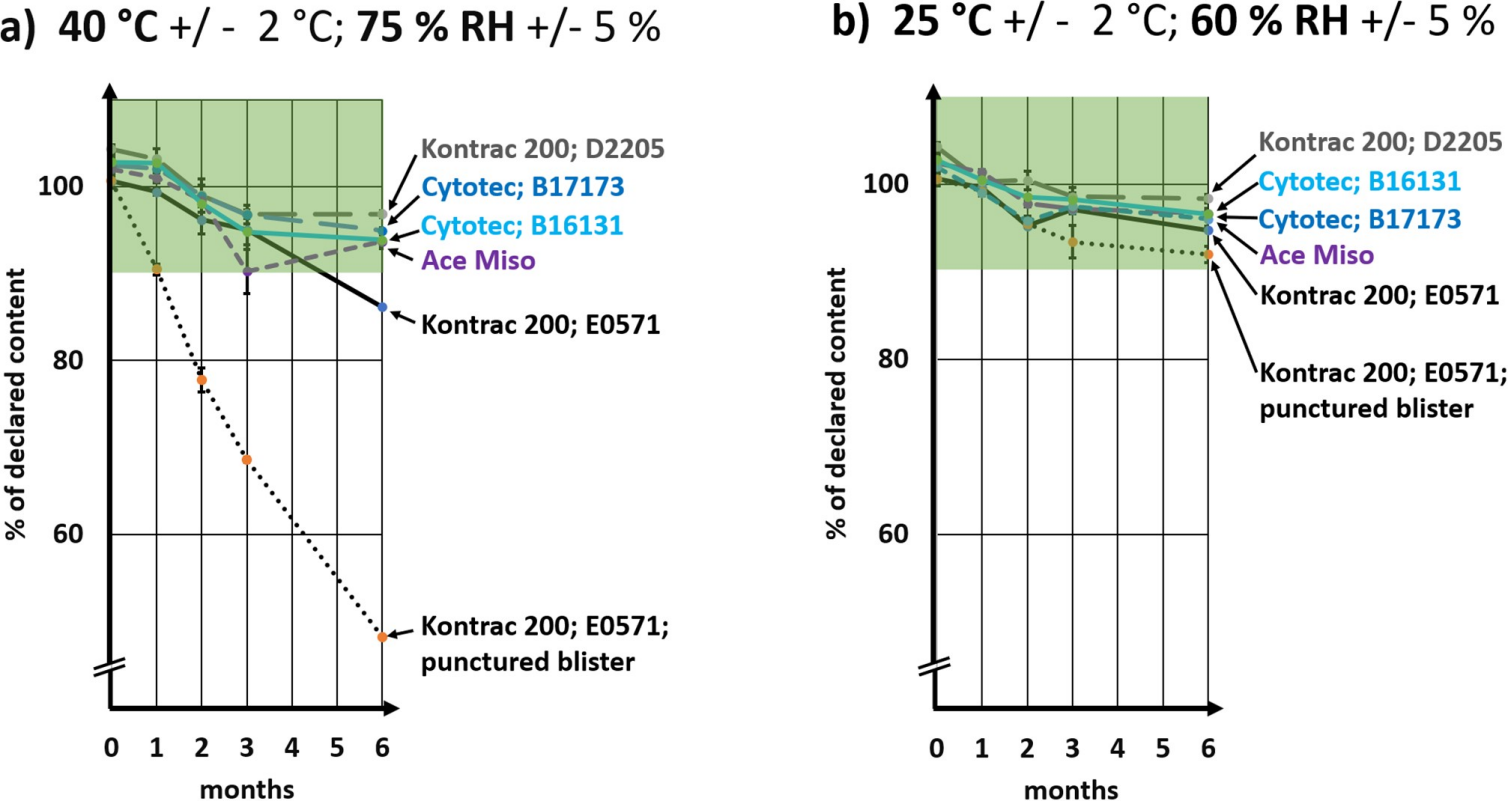
Change of misoprostol content in tablets store for 6 months a) at 40°C and 75% relative humidity (RH) and b) at 25°C and 60% RH. Error bars show standard deviation. The International Pharmacopeia requires a content of misoprostol between 90–110% of the declared amount. This range is marked in the figure.

According to the current ICH/WHO guidelines for stability testing of finished pharmaceutical products [26, 31], accelerated stability testing of medicines which are labelled for storage below 30 °C has to be performed at 40 °C and 75% RH for six months. All five collected batches of misoprostol tablets were tested under these conditions. The results are depicted in Fig 2a, and the exact misoprostol amounts determined at each time point, together with the standard deviation of the measurements, are listed in S1 Table. As visible in Fig 2a, both batches of the originator brand as well as the batch of the WHO-prequalified product remained in specifications over the entire six months testing period, i.e. their misoprostol content remained in the range of 90–110% of the declared amount. However, of the two batches of the product which had been produced in a non-SRA country and which had not been WHO-prequalified, only one batch remained in specifications, while the other batch showed a final content of 86.2% of the declared amount, which is out of specifications. The decrease of the misoprostol content of this sample was 14.5% over 6 months, which is significantly more that the decrease observed in the originator and the WHO-prequalified samples (decrease over 6 months: 8.9%, 7.4% and 8.3%, respectively; all p < 0.0001), and also significantly more than in the other investigated batch from the same manufacturer (7.5% decrease over 6 months; p < 0.0001). Contrary to expectations, the failing batch was the one with the longer remaining shelf life (Table 1), indicating that its different stability was not due to the age of the sample, but possibly due to batch-to-batch differences in the manufacturing of this product, or due to different storage conditions of the two batches prior to sample collection.

### Effect of damaged blisters

**Fig 3:**
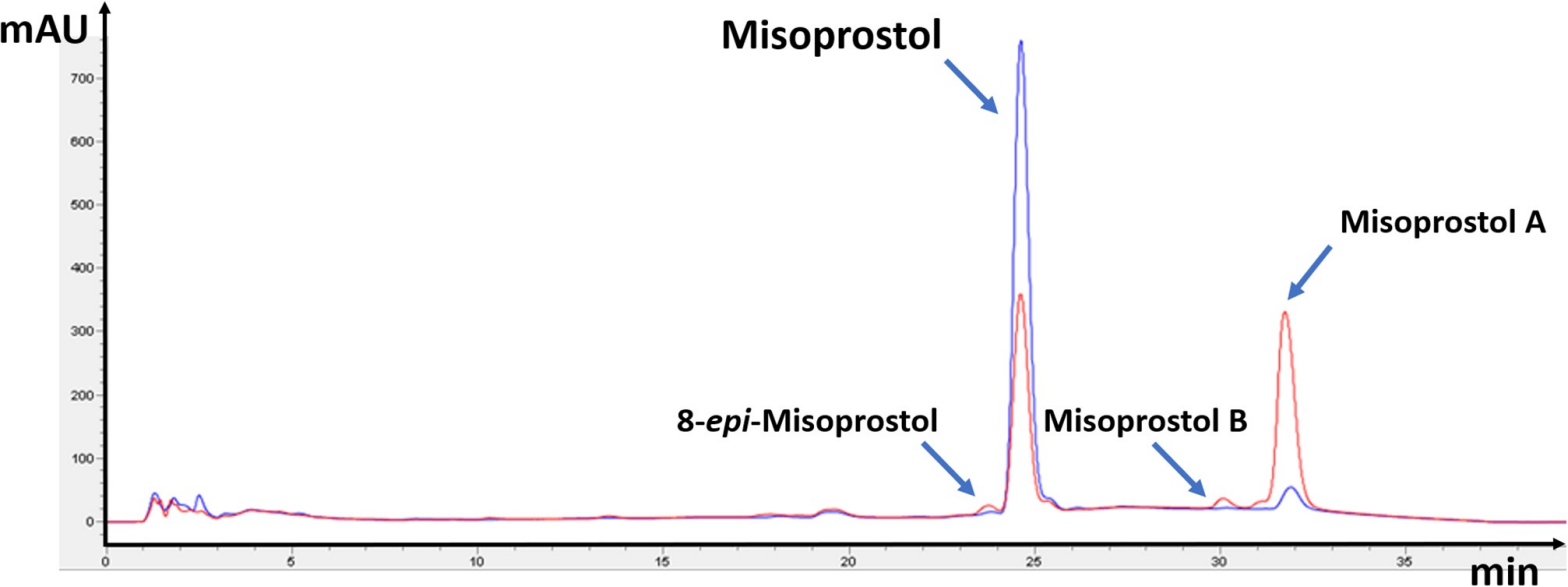
HPLC analysis of misoprostol tablets stored in damaged blisters at 40 °C and 75% RH for six months (red line). KONTRAC 200 tablets of Fourrts (India), batch E0571 (see Table 1) were used for this experiment. Tablets of the same batch stored in intact blisters at 25 °C and 60% RH for six months are shown as comparison (blue line). HPLC analysis for related substances was carried out according to Kahsay et al. [35].

All investigated products were correctly packaged in double-sided aluminium blisters. In order to investigate the importance of the intactness of the primary packaging for the stability of the misoprostol tablets, the blister strips of one sample were intentionally damaged by puncturing a single hole of approximately 1 mm diameter into each alveolus of the blisters (S1 Fig), allowing access of air and humidity to the tablets. The sample in these punctured blisters was investigated in parallel to the samples with the intact blisters. As expected, damage to the primary packaging had a strong detrimental influence on stability (Fig 2a and S1 Table): already after two months at 40 °C and 75% RH, the misoprostol content was out of specifications, and after six months the remaining amount of misoprostol was as low as 48.2% of the declared content. HPLC analysis of this sample for related substances according to Kahsay et al. [35] clearly showed the decrease of the misoprostol content and the concomitant increase of the typical degradation products of misoprostol, i.e. misoprostol A and, to a smaller extent, misoprostol B and 8-*epi*-misoprostol (Fig 3).

### Stability at long-term storage conditions

Parallel to the accelerated stability testing at 40°C and 75% RH, a control experiment was conducted at 25 °C and 60% RH, which represents the long-term storage condition for non-refrigerated products in climatic zone II [26, 31]. As shown in Fig 2b and in S1 Table, the assay results of all five investigated batches remained in specifications under these conditions for the six months testing period. Even the sample with the punctured blisters remained in specifications, though it showed the lowest final misoprostol content of all investigated samples (Fig 2b).

Also at 25 °C and 60% RH, a decline of the misoprostol content over six month was visible (Fig 2b). Assuming that misoprostol degradation followed first-order kinetics [22], i.e. with a linear relationship between the logarithm of the content and time, we calculated from the measurements depicted in Fig 2b the expected misoprostol content at the time point two years after the date of manufacture upon continued storage at 25 °C and 60% RH. This calculation predicted that four of the investigated batches would show contents between 90.2 and 96.6% of the declared amount, i.e. would remain in specifications for assay during that time period. In contrast, the batch which had failed assay testing after accelerated stability testing (KONTRAC 200, batch E0571) was predicted to show a content of 87.3% at the time of its expiry date, which would be out of specifications. Results of such extrapolations need to be evaluated with care, but still they indicate that the differences in stability observed at 25°C were consistent with those observed at 40°C.

### Dissolution testing

All collected batches, as well as the sample with the intentionally punctured blisters, were also tested for dissolution of the active pharmaceutical ingredient (API). The International Pharmacopoeia requires that at least 80% of the declared amount of misoprostol dissolves under the defined conditions (see Methods). As shown in Table 2, all five investigated batches perfectly complied with this requirement, showing nearly complete dissolution of the API. Storage in intact blisters for six months at 40°C and 75% RH resulted only in moderate decreases of the dissolution values (Table 2). These mainly reflected the loss of the API content described above, while the dissolved percentage of the API showed only small changes over six months (Table 2, right columns). All five batches remained within specifications, but the batch of the non-SRA product which had failed assay testing after storage under these conditions showed a final dissolution value of 81.3%, passing the 80% threshold of the pharmacopoeia only by a narrow margin.

**Table 2:** Dissolution testing results of misoprostol tablets stored at two different conditions, 0 and 6 months.

		Dissolution (% of declared content)				Dissolution (% of assay determined at the respective month [see S1 Table])	
Storage condition	Sample	Month 0	RSD	Month 6	RSD	Month 0	Month 6
**40°C** +/ - 2°C	Kontrac 200, batch E0571	99.2	0.98%	81.3	2.81%	98.6	94.3
	Kontrac 200, batch D2205	100.4	5.76%	95.1	1.72%	96.3	98.2
	Ace Miso	99.7	2.59%	90.8	1.57%	97.7	96.9
	Cytotec, batch B16131	104.0	3.03%	92.3	1.86%	101.1	98.3
**75% RH** +/- 5%							
	Cytotec, batch B17173	99.9	2.82%	91.0	2.26%	97.5	95.8
	Kontrac 200, batch E0571; punctured blister	99.2	0.98%	42.4	3.87%	98.6	87.9
**25°C** +/- 2°C	Kontrac 200, batch E0571	see above		90.6	2.93%	see above	95.6
	Kontrac 200, batch D2205			93.1	2.21%		94.6
	Ace Miso			93.0	2.93%		96.8
	Cytotec, batch B16131			93.1	1.97%		96.3
**60% RH** +/- 5%							
	Cytotec, batch B17173			94.6	1.88%		97.9
	Kontrac 200, batch E0571; punctured blister			89.7	2.46%		97.5

RSD: relative standard deviation. RH: relative humidity.

The sample with the punctured blisters clearly failed dissolution specifications after six months at 40°C and 75% RH, showing 42.4% dissolution of the declared API amount. Assay testing had shown a final API content of 48.2% of the declared amount. Therefore, storage in damaged blisters at these conditions strongly reduced the API content, and to some extent also reduced the dissolved percentage of the remaining API (Table 2).

After storage at 25°C and 60% RH, all tested samples, including the one with the punctured blisters, still complied with the specifications of the pharmacopeia for dissolution of misoprostol (Table 2).

## Discussion

The results of the present study confirm that the limited stability of misoprostol represents a problem for safeguarding the quality of misoprostol tablets especially in countries with hot and humid climates. This problem can be reasonably managed by appropriate packaging and by professional formulation of the tablets. The two investigated batches of the originator product, as well as the WHO-prequalified product, were found to remain within the specifications of the International Pharmacopoeia over six months of accelerated stability testing at 40°C and 75% RH, despite the fact that already 11, 16 and 19 months had elapsed since their date of manufacture when they entered stability testing, respectively. The WHO-prequalified product even exceeded its expiry date 2 months before the end of the stability test, and still conformed to specifications both in assay and in dissolution at the end of the six-months testing period. This may be seen as a further example that WHO-prequalification is a reliable assurance of the good quality of medicines. However, of the two tested batches of a product without WHO-prequalification and produced in a country without stringent regulatory authority (SRA), one batch was clearly out of specifications at the end of the accelerated stability testing. The other batch of this manufacturer showed much less degradation during accelerated stability testing. The clearly different results between the two batches may raise doubts about the batch-to-batch consistency in the manufacturing of this product.

In this study, misoprostol tablets were included in the stability test only if they were within specifications at the beginning of the test. One brand of misoprostol tablets collected in Rwanda (packaged in double-sided aluminium blisters; manufactured in a country without SRA; not WHO-prequalified) had to be excluded due to extreme deviations from specifications in the assay. Furthermore, Hall [25], Anyakora et al. [11] and Hagen et al. [12] have reported insufficient misoprostol contents in many misoprostol preparations collected in LMICs. Therefore, quality and/or stability problems are certainly not a rare observation in misoprostol tablets circulating in LMICs.

The observation that several batches investigated in this study remained in specifications during accelerated stability testing does not prove the absence of stability problems. The current ICH/WHO guidelines [26, 31] require not only that the investigated preparations remain in specifications over the six-months testing period, but also that the content of the API does not change by more than 5%. None of the five investigated batches complied with this criterion in our accelerated stability test. This result has to be interpreted with care, since the products had been stored for extended periods of time, at unknown conditions, before the stability test was conducted. Therefore, the present results do not prove that the preparations failed their stability requirements at the time when they left the manufacturing company. Nevertheless, the data reported in the present study confirm that the stability of misoprostol tablets is problematic. This is also stated in the assessment report of the WHO prequalification programme for the misoprostol tablets produced by Acme (Table 1): “The product is chemically not very stable; the data show an increase of degradation with time at accelerated and long term storage conditions, though within justified limits” [38].

In the present study, a decrease of the misoprostol content was observed both at 40°C and 75% RH and at 25°C and 60% RH (Fig 2). An investigation of the storage conditions of misoprostol tablets in health facilities in Malawi had shown mean kinetic temperatures at the different storage sites ranging from 21.4°C to 31.0°C [12]. In view of these observations, a reduction of the stated shelf-life of the originator brand (Table 1) from three to two years may be advisable, especially for those batches which are exported to countries with hot and humid climates. Also the omission of a storage requirement on the packaging of the originator product marketed in Rwanda is surprising and should be corrected by the manufacturer.

Manufacturers are aware of the stability problems of misoprostol. They frequently manufacture tablets with an initial content of more than 100% of the stated amount, to ensure that the misoprostol content of their preparations does not fall below the pharmacopeial limit of 90% of the declared amount within their shelf-life. This was observed in the present study: the content of all preparations at the beginning of the stability testing exceeded 100% of the stated amount, despite the time which had elapsed since manufacture. The data reported by Hall [25] showed that out of 215 samples of misoprostol tablets which were investigated at an age between zero and three years after their date of manufacture, approximately 43% contained between 100–110% of the stated content, and 5% even exceeded the pharmacopeial limit of 110% at the time of analysis. It has to be expected that the prevalence of overdosed preparations would be even higher if all of them had been investigated shortly after their date of manufacture. Also for the preparations investigated in the present study, extrapolation of the data shown in Fig 2b indicates that their misoprostol content at the time of manufacture may have exceeded the pharmacopeial limit of 110%. However, as mentioned above, results of such extrapolations need to be interpreted with care.

It is encouraging that all brands of misoprostol tablets distributed by wholesalers and government stores in Malawi and Rwanda at the time of sample collection were packaged in double-sided aluminium blisters. This may be a consequence of the report by Hall [25] that plastic-aluminium blisters are grossly inadequate to ensure stability of misoprostol tablets. According to the information listed on the website of the WHO prequalification of medicines programme [39], all three WHO-prequalified brands of misoprostol tablets are packaged in double-sided aluminium blisters for protection of the tablet against moisture, and are produced from 1:100 misoprostol dispersion in HPMC [38].

Both the originator product [40, 41] and at least one generic preparation produced in India [12, 25] are also marketed in screw-cap plastic bottles containing 60 or 100 tablets. While such bottles in principle offer good protection if also a desiccant in sufficient amount is included in the packaging [42], their use in LMICs is not advisable since proper closure of the bottle may not always be ensured and thereby access of humidity may severely affect the quality of the tablets. A preparation packaged in such bottles found in Malawi contained only 48.8% of the misoprostol amount declared on the label 29 months after the date of manufacture [12].

For misoprostol tablets packaged in double-sided aluminium blisters, the intactness of the blister is of principal importance for the stability. This was reported by Berard et al. [23] and has been clearly confirmed in the present study. If the blister was damaged, we observed more than 50% loss of misoprostol within 6 months at 40°C and 75% RH. The velocity of the degradation was strongly dependent on the environmental conditions: at 25°C and 60% RH, only 8.7% loss occurred within 6 months. 1.5% loss was observed in the first month under these conditions. This is somewhat different from the time pattern reported by Berard et al. [23], who had investigated another brand of misoprostol tablets for a period of one month after complete removal from their blister packs. At 25°C and 60% RH, these authors reported a loss of misoprostol content of approximately 10% within the first week, followed (somewhat surprisingly) by a loss of only about 0.5% per week in the following three weeks.

The results of our study show that unprotected storage of misoprostol tablets for extended periods at high temperature and humidity is extremely detrimental, while unprotected storage for one month at 25°C and 60% RH only had a small effect on the misoprostol content. Nevertheless, in view of the instability of misoprostol, storage outside of intact blisters should be avoided, even for short periods.

The key stability problem of misoprostol tablets is the degradation of the API, especially in the absence of proper protection from humidity. In contrast, dissolution of misoprostol was hardly affected during accelerated stability testing in this study, and even unprotected storage did not affect dissolution of the remaining amount of misoprostol very strongly (see Table 2).

### Limitations of this study

This study investigated only those brands and batches of misoprostol tablets which were available at government medical stores and pharmaceutical wholesalers in Malawi and Rwanda during the time of sample collection, and may not be representative for other brands or batches. Furthermore, while this approach to sample collection provides a realistic picture of the stability of the products which were distributed in these countries at that time, it does no give a precise picture of the condition of the samples at the time when they left the manufacturing companies. Differences in the results obtained for different brands and batches may be influenced by the different age of samples at time of analysis, and by their different storage condition prior to collection.

## Conclusions

The stability of misoprostol tablets is problematic and must be ensured by intact, good-quality double-sided aluminium blisters and by appropriate manufacturing using HPMC as stabilizing agent. Misoprostol tablets of very different quality and stability are on the market, and careful supplier qualification is required in the procurement process. In doubt, procurement should be restricted to WHO-prequalified products, and/or products manufactured under supervision of a stringent regulatory authority. In the prevention and treatment of PPH, misoprostol tablets offer the advantage over oxytocin injections that they do not need parenteral administration. However, the notion that misoprostol tablets present fewer stability problems than oxytocin injections may be misleading, especially in hot and humid climates.

## Supporting information

S1 Fig Intentionally punctured blister of misoprostol tablets (Kontrac 200, batch E0571). Needle punctures are highlighted by arrows.
(DOCX)

S1 Table Assay testing results of misoprostol tablets stored at two different conditions over 6 months. (DOCX)

S2 Table Intermediate precision of misoprostol assay and dissolution. (DOCX)
