# Supplementary material for: Stability of misoprostol tablets collected in Malawi and Rwanda: Importance of intact primary packaging
Source: PLoS One. 2020 Sep 2;15(9):e0238628. doi: 10.1371/journal.pone.0238628 (PMC7467217; doi:10.1371/journal.pone.0238628)
Supplement: S1 Fig — Needle punctures are highlighted by arrows. (DOCX) [file pone.0238628.s001.docx]

**
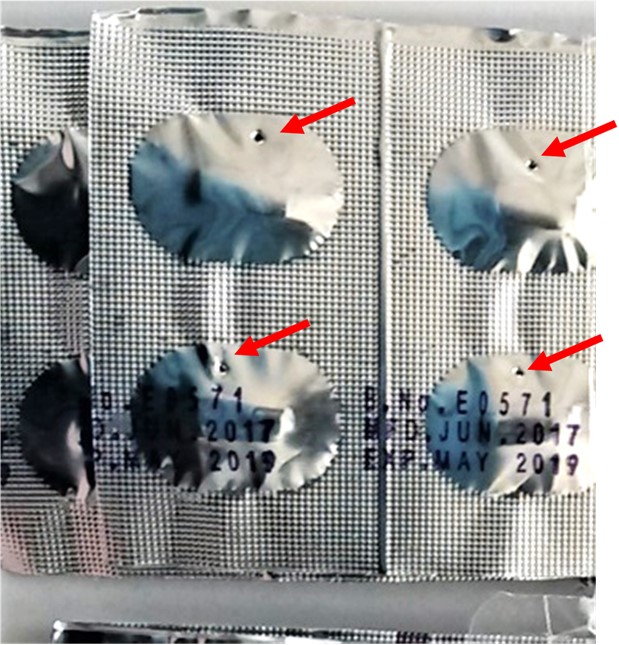
**

**S1 Fig: Intentionally punctured blister of misoprostol tablets (Kontrac 200, batch E0571). Needle punctures are highlighted by arrows.**
