# Supplementary material for: Stability of misoprostol tablets collected in Malawi and Rwanda: Importance of intact primary packaging
Source: PLoS One. 2020 Sep 2;15(9):e0238628. doi: 10.1371/journal.pone.0238628 (PMC7467217; doi:10.1371/journal.pone.0238628)
Supplement: S1 Table — (DOCX) [file pone.0238628.s002.docx]

**S1 Table: Assay testing results of misoprostol tablets stored at two different conditions over 6 months**

|  |  | **Misoprostol assay (% of declared content)** | | | | | | | | | |
| --- | --- | --- | --- | --- | --- | --- | --- | --- | --- | --- | --- |
| **Storage condition** | **Sample** | **Month 0** | **RSD** | **Month 1** | **RSD** | **Month 2** | **RSD** | **Month 3** | **RSD** | **Month 6** | **RSD** |
| **40°C**  +/- 2°C  **75% RH**  +/- 5% | **Kontrac 200, batch E0571** | 100.7 | 0.82% | 99.4 | 0.42% | 96.2 | 1.71% | 95.0 | 1.85% | 86.2 | 0.15% |
|  | **Kontrac 200, batch D2205** | 104.3 | 0.43% | 103.2 | 0.14% | 99.0 | 1.22% | 96.8 | 0.50% | 96.8 | 0.42% |
|  | **Ace Miso** | 102.0 | 0.44% | 101.0 | 0.58% | 98.4 | 0.60% | 90.3 | 2.81% | 93.7 | 0.85% |
|  | **Cytotec, batch B16131** | 102.8 | 0.41% | 102.7 | 1.56% | 98.1 | 0.42% | 94.8 | 2.07% | 93.9 | 0.72% |
|  | **Cytotec, batch B17173** | 102.4 | 1.13% | 102.1 | 0.20% | 99.0 | 1.91% | 96.8 | 1.12% | 94.9 | 0.34% |
|  | **Kontrac 200, batch E0571; punctured blister** | 100.7 | 0.82% | 90.5 | 0.69% | 77.8 | 1.75% | 68.6 | 0.47% | 48.2 | 0.24% |
|  |  |  |  |  |  |  |  |  |  |  |  |
| **25°C**  +/- 2°C  **60% RH**  +/- 5% | **Kontrac 200, batch E0571** | see above | | 99.6 | 1.28% | 95.2 | 1.12% | 97.1 | 1.32% | 94.8 | 0.20% |
|  | **Kontrac 200, batch D2205** |  |  | 100.3 | 1.16% | 100.4 | 1.03% | 98.6 | 1.09% | 98.4 | 0.48% |
|  | **Ace Miso** |  |  | 99.0 | 0.21% | 95.8 | 0.62% | 97.5 | 0.93% | 96.0 | 0.87% |
|  | **Cytotec, batch B16131** |  |  | 100.5 | 0.37% | 98.6 | 0.17% | 98.3 | 0.77% | 96.6 | 0.62% |
|  | **Cytotec, batch B17173** |  |  | 101.4 | 0.29% | 97.8 | 0.39% | 97.2 | 0.27% | 96.6 | 0.14% |
|  | **Kontrac 200, batch E0571; punctured blister** |  |  | 99.2 | 0.49% | 95.5 | 0.45% | 93.4 | 2.01% | 92.0 | 1.04% |

RSD: relative standard deviation. RH: relative humidity.
