# Supplementary material for: Stability of misoprostol tablets collected in Malawi and Rwanda: Importance of intact primary packaging
Source: PLoS One. 2020 Sep 2;15(9):e0238628. doi: 10.1371/journal.pone.0238628 (PMC7467217; doi:10.1371/journal.pone.0238628)
Supplement: S2 Table — (DOCX) [file pone.0238628.s003.docx]

**S2 Table: Intermediate precision of misoprostol assay and dissolution**

| **Intermediate Precision Calibration Curve Assay Misoprostol** | | | | | | | | | | | | |
| --- | --- | --- | --- | --- | --- | --- | --- | --- | --- | --- | --- | --- |
| **Reference concentration (µg/ml)** | Mean AUC (mAU*s) Month 0 | *RSD* | Mean AUC (mAU*s) Month 1 | *RSD* | Mean AUC (mAU*s) Month 2 | *RSD* | Mean AUC (mAU*s) Month 3 | *RSD* | Mean AUC (mAU*s) Month 6 | *RSD* | **Mean AUC (mAU*s) all months** | **RSD** |
| 25 | 1706.08 | *0.76%* | 1669.21 | *0.21%* | 1582.35 | *1.45%* | 1597.30 | *0.41%* | 1656.25 | *0.13%* | **1642.24** | **3.14%** |
| 20 | 1308.22 | *0.64%* | 1292.34 | *0.58%* | 1299.18 | *0.34%* | 1315.76 | *0.36%* | 1306.89 | *0.49%* | **1304.48** | **0.69%** |
| 15 | 1044.85 | *0.50%* | 975.97 | *0.47%* | 975.27 | *0.52%* | 1006.14 | *0.25%* | 981.14 | *0.60%* | **996.67** | **2.98%** |
| 10 | 708.98 | *0.24%* | 651.95 | *0.70%* | 700.70 | *0.75%* | 671.94 | *1.07%* | 645.61 | *0.29%* | **675.84** | **4.20%** |
| 5 | 353.04 | *1.06%* | 325.19 | *0.62%* | 377.79 | *1.35%* | 372.71 | *0.53%* | 372.04 | *0.30%* | **360.15** | **6.02%** |
|  |  |  |  |  |  |  |  |  |  |  |  |  |
| **Intermediate Precision Calibration Curve Dissolution Misoprostol** | | | | | | | | | | | | |
| **Reference concentration (µg/ml)** | Mean AUC (mAU*s) Month 0 | *RSD* | Mean AUC (mAU*s) Month 1 | *RSD* | Mean AUC (mAU*s) Month 2 | *RSD* | Mean AUC (mAU*s) Month 3 | *RSD* | Mean AUC (mAU*s) Month 6 | *RSD* | **Mean AUC (mAU*s) all months** | **RSD** |
| 0.6 | 94.37 | *3.16%* | 88.62 | *1.34%* | 92.06 | *0.15%* | 91.01 | *4.57%* | 89.74 | *1.72%* | **91.52** | **2.61%** |
| 0.4 | 61.13 | *4.35%* | 57.88 | *1.12%* | 60.58 | *2.55%* | 59.28 | *0.97%* | 60.26 | *1.73%* | **60.13** | **1.46%** |
| 0.32 | 49.90 | *9.32%* | 45.88 | *1.17%* | 48.36 | *2.33%* | 45.60 | *1.77%* | 47.13 | *2.72%* | **47.43** | **4.34%** |
| 0.24 | 34.61 | *5.22%* | 33.66 | *3.49%* | 34.07 | *4.63%* | 33.59 | *1.40%* | 36.97 | *0.55%* | **33.98** | **1.37%** |
| 0.1 | 11.28 | *9.05%* | 13.40 | *5.32%* | 12.80 | *4.30%* | 13.23 | *2.08%* | 15.01 | *4.07%* | **12.68** | **7.61%** |

AUC: area under the curve. RSD: relative standard deviation. Mean AUC calculated based on five measurements for reference concentration 20 µg/ml, and three measurements for all other reference concentrations.
